# When Fever Hides Fire: A Systematic Review of Clinical and Digital Approaches to Early Recognition of Pediatric Sepsis

**DOI:** 10.7759/cureus.116168

**Published:** 2026-09-13

**Authors:** Mohammed Elmostafa Khalid Abdulrahman, Ola Yassir Osman Abdalmagid, Abdulbagie Musaad Algylani Almardy, Osman Osama Osman Mohamed Elhassan, Ibraheem Abu Haltem, Asim Ahmed, Nahla Bakri Ageeb Babekr

**Affiliations:** 1 Department of Pediatrics, King's College Hospital London - Dubai, Dubai, ARE; 2 Department of Pediatric Medicine, Dongola Specialized Hospital, Dongola, SDN; 3 Department of Pediatrics, Maternity and Children Hospital, Buraidah, SAU; 4 Faculty of Medicine and Surgery, Omdurman Islamic University, Omdurman, SDN; 5 Department of General Medicine, Al-Aman Medical Center, Amman, JOR; 6 Faculty of Medicine and Surgery, University of Gezira, Wad Madani, SDN; 7 Department of Pediatrics, Sudan Medical Specialization Board, Khartoum, SDN

**Keywords:** clinical decision support, early diagnosis, machine learning, pediatric sepsis, sepsis screening

## Abstract

Early recognition of pediatric sepsis remains challenging because clinical presentation, sepsis definitions, and recognition approaches vary across healthcare settings. This systematic review evaluated clinical and digital strategies for the early recognition of pediatric sepsis, including structured screening tools, electronic alerts, prediction models, machine-learning approaches, clinical decision support, and recognition pathways. Diagnostic criteria were considered separately as reference frameworks for classifying established sepsis and interpreting the performance of recognition tools. PubMed and Scopus were systematically searched, eligible studies were independently screened, data were extracted, and methodological quality and risk of bias were assessed using design-appropriate tools. Because of substantial clinical and methodological heterogeneity, the evidence was synthesized narratively. A total of 13 studies were included. Several clinical and digital approaches generally performed well in ruling out sepsis among low-risk children; however, their ability to identify true cases and limit unnecessary alerts varied according to alert thresholds, clinical settings, and the sepsis definition or reference standard used. No single approach was consistently superior for early recognition. Automated screening appeared most useful when integrated with structured clinical assessment rather than used independently. Differences in pediatric sepsis definitions substantially influenced reported performance, and implementation studies more consistently demonstrated improvements in recognition and care processes than in patient outcomes. The available evidence was limited by heterogeneous methods, imperfect reference standards, insufficient external validation, and risk of bias. Clinical and digital recognition systems may support earlier identification of children with suspected infection who require further assessment; however, current evidence does not establish a universally superior approach. Further prospective multicenter studies are needed to determine which strategies provide meaningful clinical benefit across diverse healthcare settings.

## Introduction and background

Sepsis remains a major cause of preventable morbidity and mortality in children worldwide, with the burden falling disproportionately on younger children and populations in resource-limited settings [1]. Beyond acute mortality, pediatric sepsis is associated with organ dysfunction, prolonged critical illness, and important effects on physical, cognitive, and emotional functioning among survivors, making timely recognition a central component of pediatric emergency and critical care [2].

The conceptual definition of pediatric sepsis has also evolved. The 2024 International Consensus Criteria replaced the previous systemic inflammatory response syndrome-based framework with the Phoenix Sepsis Score, which defines sepsis through infection-associated life-threatening organ dysfunction [3]. The Phoenix criteria classify established organ dysfunction and are not designed as an initial screening tool. In practice, early screening tools identify children who may be at risk and require further clinical assessment, whereas the Phoenix criteria classify infection-associated organ dysfunction once it is established. Therefore, an alert triggered before organ dysfunction becomes established may represent a useful early warning rather than a false-positive finding. Contemporary pediatric sepsis guidelines continue to emphasize early identification and structured clinical assessment before or alongside definitive organ-dysfunction classification [4].

Previous literature shows why early recognition remains challenging. A systematic review by Menon et al. found strong associations between measures of organ dysfunction and pediatric sepsis outcomes but noted that pre-intensive-care populations, where early recognition is most relevant, were substantially underrepresented [5]. This limits the applicability of the findings to emergency departments and general pediatric wards, where children may present earlier with less severe or less specific signs and where recognition tools may perform differently. A subsequent scoping review of pediatric emergency departments identified 89 studies evaluating clinical parameters, biomarkers, alerts, and other identification methods [6]. However, differences in populations, study designs, reference standards, and outcome definitions prevented fair comparison between the approaches; this did not mean that all approaches were unreliable.

Digital approaches have expanded this evidence base. Sanchez-Pinto et al. described the increasing use of electronic health record-based clinical decision support, predictive analytics, and artificial intelligence in pediatric sepsis, while highlighting the limited rigorous evaluation of these approaches and the concentration of digital innovation in high-resource healthcare systems [7]. A scoping review of pediatric sepsis prediction technologies found that most studies were single-center, frequently involved low-prevalence outcomes, used variable prediction horizons and endpoints, and inconsistently addressed workflow integration and human factors [8]. More recently, Driver et al. reviewed automated electronic sepsis alerts and found improvements in some care-process measures, but their effects on clinical outcomes remained uncertain because of heterogeneity and confounding across the available studies [9].

Despite this growing literature, important gaps remain. Previous reviews generally examined individual recognition approaches separately, including emergency-department identification methods, prediction technologies, digital solutions, and automated alerts [6-9]. In contrast, the present review considers clinical and digital recognition strategies together and examines their diagnostic and predictive performance, clinical integration and implementation, effects on care processes, and patient outcomes. Comparisons are further complicated by differences in sepsis definitions and reference standards, while evidence on external validation, clinician integration, implementation effects, and patient-level benefit remains incomplete.

Therefore, this systematic review evaluated clinical and digital approaches to the early recognition of pediatric sepsis in children aged 29 days to 18 years. We examined their diagnostic and predictive performance, their integration with clinician assessment, and their effects on recognition time, care processes, and patient outcomes. We also assessed how differences in sepsis definitions, methodological quality, risk of bias, and generalizability influenced the interpretation of the evidence.

## Review

Methods

Study Design and Reporting

This systematic review evaluated clinical and digital approaches for the early recognition of sepsis and septic shock in children and was conducted and reported in accordance with the Preferred Reporting Items for Systematic Reviews and Meta-Analyses (PRISMA) 2020 statement [10]. The review was retrospectively registered in the SciMENA Protocol Registry on August 29, 2026 (registration number: SciPro-00000230; https://scimena.com/protocol/registry/SciPro-00000230). The literature search and initial screening had commenced before registration. The eligibility criteria, screening process, data extraction approach, risk-of-bias assessment, and synthesis methods are reported transparently.

Information Sources and Search Strategy

The formal literature search was conducted in PubMed/MEDLINE (Medical Literature Analysis and Retrieval System Online) and Scopus, with the final database search performed on June 18, 2026. PubMed/MEDLINE was selected to capture the core biomedical and clinical literature, while Scopus was used to broaden coverage across multidisciplinary, informatics, and technology-focused journals. Two complementary PubMed searches, comprising a core search and a supplementary search, were conducted to maximize sensitivity, and Scopus was searched to identify additional eligible records.

The search strategy combined terms related to the pediatric population, sepsis and septic shock, and early clinical or digital recognition. Search concepts included children, pediatric, paediatric, infant, adolescent, sepsis, severe sepsis, septic shock, screening, recognition, early identification, diagnosis, triage, early-warning systems, prediction models, algorithms, electronic alerts, electronic health records, clinical decision support, machine learning, and artificial intelligence. Database-specific syntax was adapted to each information source. The exact core PubMed search strategy, together with the available documentation for the supplementary PubMed and Scopus searches, is provided in Supplementary Table 8. The original literal strings for the supplementary PubMed and Scopus searches were not retained, and this limitation is reported transparently.

The search-strategy framework and database yields are summarized in Table 1.

**Table 1:** Search strategy framework and database yield The search was designed around pediatric population terms, sepsis and septic shock terms, and early clinical or digital recognition concepts. Database-specific syntax was adapted to PubMed/MEDLINE and Scopus. AI, artificial intelligence; CDS, clinical decision support; EHR, electronic health record; ML, machine learning.

Information Source	Search Component	Search Coverage	Last Search Date	Records Retrieved
PubMed/MEDLINE	Core search	Pediatric population, sepsis or septic shock, and early recognition, screening, and diagnosis terms	June 18, 2026	n = 1,176
PubMed/MEDLINE	Supplementary search	Complementary sensitivity search expanding prediction models, algorithms, electronic alerts, EHRs, CDS, ML, and AI terms	June 18, 2026	n = 2,277
Scopus	Complementary database search	Database-adapted equivalent of the pediatric sepsis and clinical or digital recognition concept set	June 18, 2026	n = 100
Total before deduplication	—	All records identified across the three search runs	—	n = 3,553

Record Management and Deduplication

All retrieved references were managed using Zotero (Corporation for Digital Scholarship, Vienna, Virginia, United States) and Microsoft Excel (Microsoft Corporation, Redmond, Washington, United States). Zotero was used for reference management and initial duplicate identification, while structured Microsoft Excel sheets were used for screening and eligibility tracking. Duplicate records were identified using PMID, DOI, and title matching, with manual verification where required. Only unique records proceeded to title and abstract screening.

Eligibility Criteria

Studies were eligible if they included children aged 29 days to 18 years with suspected infection, sepsis, severe sepsis as historically defined, or septic shock in an acute-care setting. Eligible settings included pediatric emergency departments, inpatient pediatric wards, pediatric intensive care units, urgent-care facilities, and other acute-care environments. Neonates were excluded because neonatal sepsis has distinct clinical presentations, definitions, risk factors, and recognition pathways that differ from those used in older infants and children.

Eligible approaches included clinical screening tools, sepsis or early-warning scores, electronic alerts, automated algorithms, clinical decision-support systems, prediction models, machine-learning or artificial-intelligence approaches, diagnostic criteria, structured clinician assessment, parent or healthcare professional concern, and structured recognition pathways.

Studies were required to report at least one direct recognition-related outcome. Eligible outcomes included diagnostic-accuracy measures, such as sensitivity, specificity, predictive values, and likelihood ratios; prediction-model performance, including discrimination and calibration; alert and screening performance, including screening yield, false-positive or false-negative findings, missed cases, and alert burden; or recognition and immediate pathway outcomes, including diagnostic agreement, time to recognition, clinician reassessment, or escalation following an alert. An unlisted outcome was considered eligible only when it directly measured recognition or the immediate response to recognition. Outcomes related only to treatment, prognosis, or later clinical events were not sufficient for inclusion.

Patient-related exclusions included neonatal-only studies, neonatal intensive care unit studies, early- or late-onset neonatal sepsis studies, cord-blood studies, and neonatal sepsis calculators. These were excluded because neonatal sepsis has distinct risk factors, definitions, clinical presentations, and recognition pathways. Mixed-age studies were excluded when results for participants aged 18 years or younger could not be separately extracted; participants exactly 18 years old were considered eligible.

Study- and publication-related exclusions included isolated biomarker, laboratory, microbiological, or molecular diagnostic studies unless the test was evaluated as part of a clinical recognition strategy or pathway and a direct recognition-related outcome was reported. Studies combining recognition and prognosis were eligible only when recognition outcomes could be identified separately. Prognosis-only, treatment-only, epidemiological, qualitative, review, commentary, guideline, protocol, and case-report publications without eligible recognition outcomes were excluded.

The detailed inclusion and exclusion criteria are presented in Table 2.

**Table 2:** Inclusion and exclusion criteria AUC/AUROC, area under the receiver operating characteristic curve

CriteriaType	Eligibility Criteria
Inclusion	Studies that included children older than 28 days and up to 18 years.
	Participants with suspected infection, sepsis, severe sepsis as historically defined, or septic shock.
	Studies conducted in an acute-care setting.
	Pediatric emergency departments, inpatient pediatric wards, pediatric intensive care units, urgent-care facilities, and other acute-care environment settings
	Studies evaluating clinical screening tools, sepsis scores, or early-warning scores.
	Studies evaluating electronic alerts, automated algorithms, or clinical decision-support systems.
	Studies evaluating prediction models or machine-learning or artificial-intelligence approaches.
	Studies evaluating diagnostic criteria, structured clinician assessment, parent or healthcare professional concern, or structured recognition pathways.
	Studies reporting at least one direct recognition-related outcome, including sensitivity, specificity, predictive values, likelihood ratios, AUC/AUROC, model discrimination or calibration, diagnostic agreement, missed or delayed recognition, false-positive or false-negative findings, time to recognition, alert performance, or another recognition-related pathway outcome.
Exclusion	Neonatal-only studies
	Neonatal intensive care unit studies
	Studies of early- or late-onset neonatal sepsis
	Cord-blood studies
	Studies evaluating neonatal sepsis calculators
	Mixed-age studies, when results for participants aged 18 years or younger could not be separately extracted.
	Isolated biomarker studies without a clinical recognition component
	Isolated laboratory, microbiological, or molecular diagnostic studies without a clinical recognition component
	Prognosis-only studies
	Treatment-only studies
	Epidemiological studies without eligible recognition outcomes
	Qualitative studies without eligible recognition outcomes
	Reviews and commentaries
	Guidelines and protocol-only publications
	Case reports without eligible recognition outcomes

The eligibility framework is further summarized using PICO (Population, Intervention/Index Approach, Comparator/Reference, and Outcomes) in Table 3.

**Table 3:** PICO framework for eligibility and review outcomes NOTE: Because the review included diagnostic, prediction-model, implementation, and randomized study designs, the comparator or reference standard varied according to the study design. AUC/AUROC, area under the receiver operating characteristic curve; PICO, Population, Intervention/Index Approach, Comparator/Reference, and Outcomes.

PICO Element	Operational Definition
Population	Children older than 28 days and up to 18 years with suspected infection, sepsis, severe sepsis as historically defined, or septic shock in acute-care settings.
Intervention / Index Approach	Structured clinical or digital recognition strategies, including screening or early-warning tools, electronic alerts, algorithms, clinical decision support, prediction models, machine-learning or artificial-intelligence systems, diagnostic criteria, structured clinician assessment, parent or healthcare professional concern, and recognition pathways.
Comparator / Reference	The study-specific comparator or reference standard, including consensus criteria, clinician-adjudicated diagnosis, alternative recognition tools or definitions, usual care, or other prespecified clinical reference approaches, as applicable to the study design.
Outcomes	Recognition-related performance or pathway outcomes, including sensitivity, specificity, predictive values, likelihood ratios, AUC/AUROC, calibration or discrimination, diagnostic agreement, false-positive or false-negative findings, missed or delayed recognition, time to recognition, alert performance, and other recognition-related pathway outcomes.

Study Selection

Titles and abstracts were screened independently by two researchers (MEKA and NBAB). Screening decisions were concealed between reviewers until both assessments had been completed. Disagreements were resolved through discussion, and unresolved disagreements were adjudicated by AA as the third reviewer.

Following the initial broad screening stage, but before final full-text adjudication, the scope was narrowed from the wider pediatric sepsis literature to studies directly evaluating clinical or digital approaches to early recognition in children aged 29 days to 18 years. The original framework included neonates and broader evidence on biomarkers, laboratory diagnosis, blood-culture practices, diagnostic stewardship, qualitative evidence, and health-system barriers. The amended framework excluded neonatal-only studies and isolated laboratory or biomarker studies without a clinical recognition component and required a direct recognition-related outcome. This refinement changed which studies could be included and was documented in the screening log. All 286 candidate reports retained under the original framework were reassessed against the amended criteria; 266 did not meet the refined scope, leaving 20 reports for retrieval.

Restricted access was not considered a scientific exclusion criterion. Reports for which full text remained unavailable after available legal retrieval routes were classified as not retrieved and were not subjected to data extraction or risk-of-bias assessment.

Data Extraction

Data were extracted independently by two reviewers using a standardized Microsoft Excel extraction form. The two extraction records were compared after completion, and discrepancies were resolved through review of the original article and discussion. Unresolved disagreements were adjudicated by the third reviewer.

Extracted variables included author, publication year, country, study design, recruitment period, setting, sample size, age range, population characteristics, sepsis definition, reference standard, recognition strategy, index tool, thresholds, comparator, statistical methods, and recognition-related outcomes.

Where available, sensitivity, specificity, positive and negative predictive values, likelihood ratios, area under the receiver operating characteristic curve (AUROC), confidence intervals (CIs), diagnostic agreement, false-positive and false-negative findings, missed or delayed recognition, alert burden, screening yield, and time-to-recognition outcomes were extracted. Funding, conflicts of interest, methodological limitations, and potential overlap between publications were also recorded. Data not reported in the original study were not inferred.

Risk of Bias Assessment

Risk of bias was assessed according to study design. Diagnostic-accuracy and screening studies were evaluated using the Quality Assessment of Diagnostic Accuracy Studies 2 (QUADAS-2) tool [11]. Prediction-model and machine-learning studies were assessed using PROBAST+AI [12]. Non-randomized intervention and quality-improvement studies were assessed using the Risk Of Bias In Non-randomized Studies of Interventions (ROBINS-I) framework [13]. Randomized studies were evaluated using the revised Cochrane Risk of Bias 2 (RoB 2) tool, with cluster-randomized considerations applied to the stepped-wedge cluster-randomized study [14].

Assessments were performed independently by two reviewers, with disagreements resolved through discussion and third-reviewer adjudication when necessary. Domain-level risk-of-bias judgments were based on information reported in the full-text articles and were used to inform interpretation of the evidence rather than as a post hoc eligibility criterion. Studies were not excluded on the basis of methodological quality or risk-of-bias assessment.

Data Synthesis

A structured narrative synthesis was undertaken because substantial clinical and methodological heterogeneity was present across study designs, clinical settings, sepsis definitions, reference standards, recognition strategies, diagnostic thresholds, prediction time points, and reported outcomes.

Studies were grouped according to their principal recognition approach, including clinical screening and diagnostic criteria, electronic alerts and clinical decision support, prediction and machine-learning models, human clinical concern, and structured recognition or quality-improvement pathways. Diagnostic and predictive performance measures were summarized in structured tables together with recognition timing, alert burden, implementation outcomes, and risk-of-bias findings.

Meta-analysis was not performed because the included studies differed substantially in populations, settings, target conditions, sepsis definitions, reference standards, thresholds, prediction horizons, units of analysis, and reported outcomes. Pooling these results would not have produced a clinically meaningful summary estimate.

Formal reporting-bias assessment was not undertaken because the heterogeneity of study designs and outcomes precluded a common quantitative synthesis. A formal GRADE (Grading of Recommendations Assessment, Development, and Evaluation) certainty-of-evidence assessment was also not performed because the review combined diagnostic-accuracy, prediction-model, and implementation evidence with substantially different designs and outcomes. Certainty was interpreted cautiously using design-specific risk-of-bias assessments, consistency, external validation, and applicability.

Results

Study Selection

The searches identified 3,553 records: 1,176 from the core PubMed search, 2,277 from the supplementary PubMed search, and 100 from Scopus. After removal of 124 duplicates, 3,429 unique records were screened, and 3,143 were excluded at title/abstract screening, leaving 286 reports under the initial broad eligibility framework. Following the documented refinement to a clinical- and digital-recognition focus, 266 reports no longer met the amended scope, leaving 20 reports for final retrieval and eligibility assessment.

Four of the 20 reports sought for retrieval could not be obtained and were classified as not retrieved rather than scientifically excluded. The remaining 16 full-text reports were assessed for eligibility. Three were excluded because their mixed-age populations extended beyond the prespecified pediatric age range and pediatric-specific results could not be extracted separately, leaving 13 unique studies for inclusion. One companion report was linked to its primary study and was not counted separately. The complete study-selection process is shown in Figure 1.

**Figure 1:**
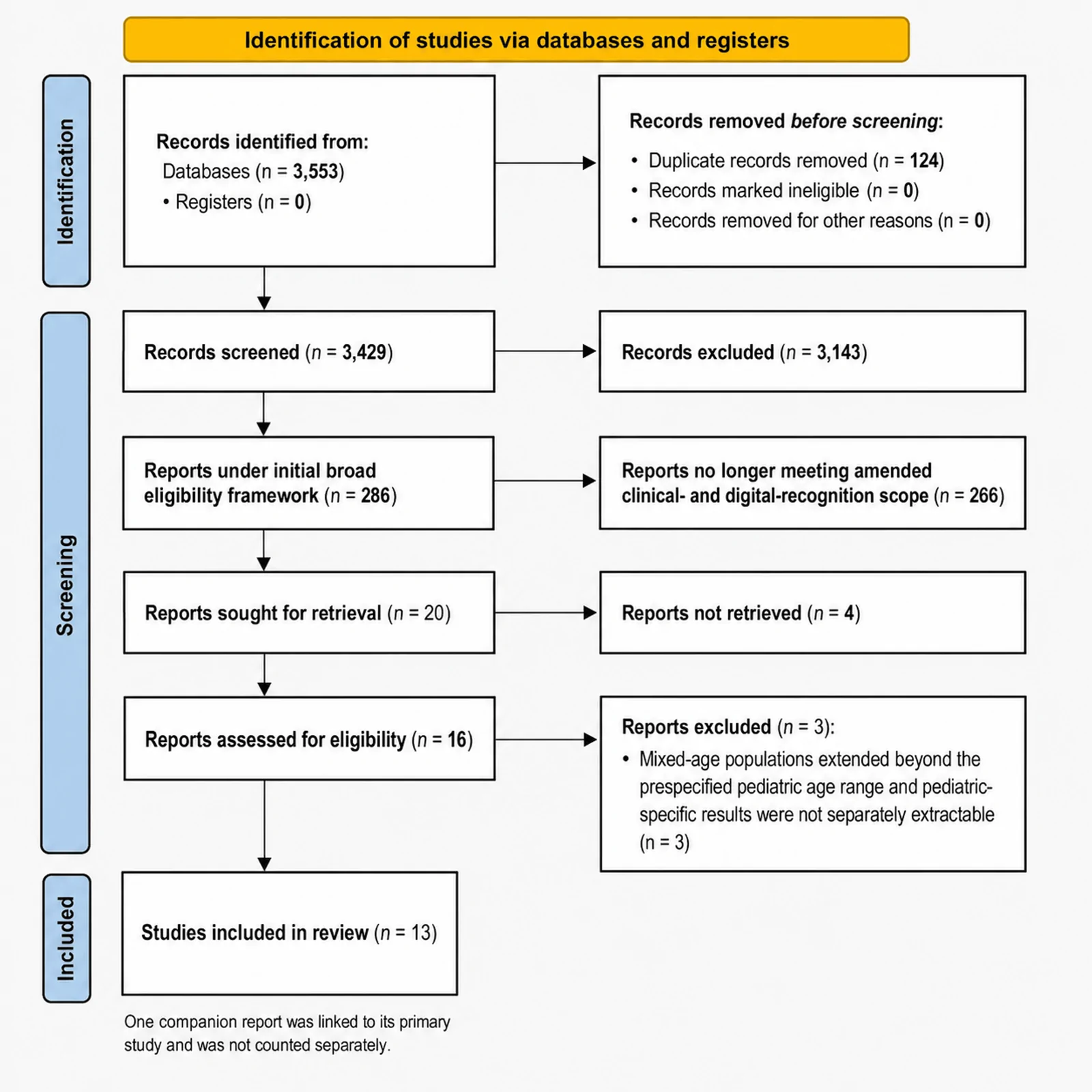
PRISMA flow diagram of study identification, screening, eligibility assessment, and inclusion. PRISMA, Preferred Reporting Items for Systematic Reviews and Meta-Analyses

Study Characteristics 

The 13 included studies were published between 2014 and 2026 [15-27]. Seven were conducted in the United States [15,19,20,22,24,25,27], two in Australia [21,26], one each in Germany [17], the United Kingdom [18], and China [23], and one was an international pediatric intensive care unit (PICU) study involving 26 countries [16]. The evidence included diagnostic-accuracy and agreement studies, screening-tool and prediction-model evaluations, electronic clinical decision support, quality-improvement programs, and one stepped-wedge cluster randomized trial. Study design, setting, and population characteristics are summarized in Table 4.

**Table 4:** Study design, setting, and population vharacteristics the included studies evaluating early recognition of pediatric sepsis ED, emergency department; EHR, electronic health record; PICU, pediatric intensive care unit; QI, quality improvement; SIRS, systemic inflammatory response syndrome

Study (Author, Year)	Country/Setting	Design	Population/Sample
Sepanski et al., 2014 [15]	United States; pediatric ED	Retrospective screening-tool development/refinement with internal split validation	n = 7,402 ED encounters; 38 gold-standard severe-sepsis cases
Weiss et al., 2015 [16]	International; 128 PICUs in 26 countries	Prospective multicenter point-prevalence agreement study	n = 6,925 screened; 706 classified as severe sepsis by ≥1 approach; age <18 years
Lamping et al., 2018 [17]	Germany; tertiary PICU	Prediction-model development with temporal validation using prospectively collected data	n = 289 episodes: 56 sepsis, 233 noninfectious SIRS; validation 32 sepsis + 103 SIRS
Gomes et al., 2021 [18]	United Kingdom; 2 pediatric EDs	Multicenter retrospective diagnostic-performance evaluation of prospectively implemented screening	n = 19,912 screened; 90 final sepsis cases
Ehwerhemuepha et al., 2021 [19]	United States; tertiary pediatric ED	Retrospective EHR machine-learning model development and internal test evaluation	n = 537,837 encounters; 295 severe sepsis, 1,102 nonsevere sepsis, 1,103 bacteremia
Yan et al., 2022 [20]	United States; pediatric ED	Retrospective diagnostic-performance cohort; pre-pandemic vs pandemic comparison	n = 111,327 encounters; 59,335 pre-pandemic and 51,990 pandemic
Gilholm et al., 2023 [21]	Australia; 12 EDs	Prospective multicenter screening/model simplification and internal validation	n = 3,473 screened; 523 sepsis, 210 organ dysfunction, 146 septic shock
Oddiri et al., 2023 [22]	United States; pediatric ED and inpatient units	Single-center longitudinal pre/post QI study	Annual pediatric sepsis population; bundle cohorts: n = 41 baseline, then 20, 18, 17, and 13
Li et al., 2025 [23]	China; tertiary PICU	Prospective single-center diagnostic-criteria comparison	n = 240 children with sepsis by 2005 criteria; 86 with shock; >28 days to ≤18 years
Dewan et al., 2020 [24]	United States; 35-bed quaternary PICU	Prospective diagnostic-performance study + pre/post QI process evaluation	n = 10,805 postimplementation patient-days; 486 sepsis-positive episodes; 424 in-PICU episodes
Scott et al., 2020 [25]	United States; tertiary ED + 5 satellite ED/urgent-care sites	Multisite uncontrolled longitudinal QI program	n = 3,843 registry patients; 932 severe-sepsis patients; 203 missed by pathway
Sever et al., 2023 [26]	Australia; 2 tertiary pediatric EDs	Prospective multicenter diagnostic-accuracy cohort	n = 533 screened; 492 included; 118 sepsis at presentation
Scott et al., 2025 [27]	United States; 4 pediatric EDs	Prospective stepped-wedge cluster randomized trial	n = 200,354 ED encounters; 1,331 eligible sepsis evaluations (979 intervention; 352 control)

Most studies were in pediatric emergency settings, whereas Weiss et al. [16], Lamping et al. [17], Li et al. [23], and Dewan et al. [24] focused on PICU populations. The studies examined both early screening strategies and criteria used to classify established sepsis-related organ dysfunction. Recognition approaches ranged from systemic inflammatory response syndrome (SIRS)-based screening and structured clinician assessment to electronic alerts, prediction and machine-learning models, clinical decision support, and organized recognition pathways. Sample sizes and units of analysis varied markedly across individual children, emergency encounters, patient-days, and clinical episodes, precluding a meaningful aggregate sample size and limiting direct comparison. The recognition approaches, reference standards or target conditions, and principal outcomes extracted from the included studies are summarized in Table 5.

**Table 5:** Recognition approaches, reference standards, and outcomes of included studies evaluating early recognition of pediatric sepsis AUC, area under the receiver operating characteristic curve; AUCPR, area under the precision-recall curve; CDS, clinical decision support; ED, emergency department; EHR, electronic health record; eST, electronic screening tool; ICU, intensive care unit; IPSCC, International Pediatric Sepsis Consensus Conference; LR+, positive likelihood ratio; LR−, negative likelihood ratio; ML, machine learning; NLR, negative likelihood ratio; NNE, number needed to evaluate; NNS, number needed to screen; NPV, negative predictive value; OR, odds ratio; PICU, pediatric intensive care unit; PLR, positive likelihood ratio; PPV, positive predictive value; pSOFA, pediatric Sequential Organ Failure Assessment; SIRS, systemic inflammatory response syndrome

Study	Recognition Approach	Reference Standard/Target	Main Outcomes Extracted
Sepanski et al. [15]	Refined electronic severe-sepsis screening algorithm using age-adjusted physiologic criteria, SIRS, organ dysfunction, and suppression rules	Infection + SIRS + organ dysfunction; blinded physician chart review	Sensitivity, specificity, PPV, NPV, AUC, false positives/negatives, alert rate, time to tool firing
Weiss et al. [16]	Attending-physician clinical recognition	2005 pediatric consensus severe-sepsis criteria; neither approach was a true gold standard	Agreement, Cohen κ, regional variation, discordant identification
Lamping et al. [17]	Conditional-inference Random Forest; 8-variable final model	IPSCC/infection criteria with blinded expert review	AUROC, sensitivity, specificity, temporal validation, cross-validation
Gomes et al. [18]	Mandatory electronic screening tool (eST) followed by clinician assessment	Sepsis treatment in ED + final inpatient clinical diagnosis	Sensitivity, specificity, PPV, NPV, LR+/LR−, under-triage, over-triage, alert yield
Ehwerhemuepha et al. [19]	XGBoost using triage variables and 6-month clinical/EHR history	Diagnosis-code-derived sepsis/severe-sepsis outcomes	AUROC, AUCPR, sensitivity, specificity, PPV, NPV, NNE
Yan et al. [20]	Continuously running modified-SIRS EHR sepsis alert	Hypotensive septic shock within 24 h using clinical/treatment criteria	Alert burden, sensitivity, specificity, PPV, NPV, missed shock, COVID subgroup
Gilholm et al. [21]	32-item pediatric sepsis pathway; simplified 16-feature model	Senior medical officer judgment + IV antibiotics; IPSCC outcomes in sensitivity analyses	AUC, calibration, sensitivity, specificity, PLR, NLR
Oddiri et al. [22]	Nurse-triggered and provider-triggered interruptive EHR alerts plus order sets, paging, education, and dashboard	SIRS + organ dysfunction; coded/quality review of severe sepsis and shock	1-h bundle compliance, alert-response compliance, missed cases
Li et al. [23]	2024 Phoenix Sepsis Score criteria	2005 pediatric consensus criteria as comparator	Diagnostic agreement, κ, sensitivity, specificity, PPV, NPV for septic shock
Dewan et al. [24]	Two-stage EHR CDS: temperature/blood-culture trigger followed by nurse perfusion assessment	Composite IPSO severe sepsis or clinician intention-to-treat; chart-reviewed severe-sepsis subset	Sensitivity, specificity, PPV, NPV, LR+/LR−, NNS, alert burden, bundle completion
Scott et al. [25]	Two-tier Sepsis Stat/Sepsis Yellow recognition pathway	Suspected infection + acute organ dysfunction; clinical recognition based on pathway/order activation	Arrival-to-recognition, antibiotic/bolus timing, ICU use, mortality, safe de-escalation
Sever et al. [26]	Parent, nurse, and physician concern measured near triage	Infection + pSOFA >0; independently adjudicated infection; IPSCC sensitivity analysis	AUC, adjusted/unadjusted ORs, incremental prediction, 48-h sepsis, PICU admission
Scott et al. [27]	Machine-learning-based clinical decision support for hypotensive septic shock	Hypotensive shock within 24 h; clinician-initiated sepsis evaluation	Guideline-concordant care, hypotensive shock, antibiotic timing, PPV, CDS adoption

Key Numerical Findings and Narrative Synthesis

Because the studies differed substantially in populations, settings, recognition strategies, reference standards, thresholds, and outcomes, results were synthesized narratively rather than pooled.

Clinical and Electronic Screening Approaches

Screening performance varied by target condition and reference standard. Sepanski et al. reported sensitivity of 97.4% and specificity of 99.5% after tool refinement, with false positives falling from 217 to 39 [15]. Gomes et al. found that the electronic screening tool alone had sensitivity of 86.7% and specificity of 87.0% [18]. Its positive predictive value (PPV), defined as the proportion of screen-positive cases that were true sepsis cases, was 2.94%. When the electronic screen was combined with subsequent clinical assessment, sensitivity increased to 90.0%, specificity to 99.4%, and PPV to 42.0%. This improvement reflected the sequential addition of clinician assessment; the study did not evaluate a separate change in the alert threshold. Yan et al. reported similar performance before and during the COVID-19 pandemic, with sensitivity of 74.1% and 72.5% and specificity of 93.2% and 94.0%, respectively [20]. However, approximately one-quarter of hypotensive shock cases occurred without an alert, indicating that automated screening did not identify all clinically important cases. Gilholm et al. reported an AUC of 0.80 for both the 32-item and simplified 16-feature tools; at 90% sensitivity, specificity was 51% [21].

Prediction Models and Machine-Learning Approaches

Lamping et al. reported an AUC of 0.78 (95% CI, 0.70-0.87) in temporal validation of an eight-variable Random Forest model; a threshold achieving 100% sepsis identification correctly classified only 28% of noninfectious SIRS episodes [17]. Ehwerhemuepha et al. evaluated 537,837 pediatric ED encounters and reported a severe-sepsis AUROC of 0.990 (95% CI, 0.985-0.995); at the selected threshold, sensitivity was 84.5%, specificity 99.0%, PPV 6.31%, and NPV 99.99% [19].

Diagnostic Definitions and Clinical Recognition

Classification differed substantially according to the sepsis definition applied. In SPROUT, 301 of 706 children (42.6%) identified by either clinician diagnosis or consensus criteria met both definitions, whereas 405 (57.4%) were identified by only one. Agreement was moderate (kappa = 0.57) and ranged from 31% to 85% across regions, a 54-percentage-point difference [16]. The study did not establish whether this variation reflected differences in clinical practice, patient severity, available resources, or interpretation of the criteria. Li et al. found poor overall agreement between the 2005 and 2024 criteria (kappa = 0.161) [23]. When the 2005 criteria were used as the comparator, the 2024 criteria had sensitivity of 57.0% and specificity of 100% for septic shock, indicating more restrictive classification rather than uniformly better diagnostic performance. These kappa estimates describe agreement between classification approaches. By contrast, Sever et al. assessed discrimination and found that concern alone had AUCs of 0.53 for parents, 0.57 for nurses, and 0.63 for physicians [26]. Adding concern to baseline clinical models produced no material improvement for sepsis at presentation, showing that concern alone was insufficient for reliable classification.

Clinical Decision Support and Recognition Pathways

Observational before-and-after studies reported substantial improvements in care-process measures. Dewan et al. reported sensitivity of 92.5%, specificity of 95.6%, PPV of 46.0%, and NPV of 99.7% for the PICU clinical decision support (CDS), while timely completion of three recommended tasks increased from 18.6% to 34.0% (P = .04), an increase of 15.4 percentage points [24]. Scott et al. reported that arrival-to-recognition time fell from approximately 50 to four minutes during a longitudinal two-tier QI program [25], and Oddiri et al. found that one-hour bundle compliance increased from 37% to 69% (P = .006) [22]. Because these programs introduced training, workflow, and behavioral changes alongside decision support, the observed improvements cannot be attributed solely to the electronic systems. In contrast, the cluster-randomized trial by Scott et al. found almost no difference in guideline-concordant care (39.0% vs 38.9%; aOR 1.07, 95% CI 0.61-1.88) and no measurable improvement in hypotensive shock or antibiotic timing [27]. All four sites retained the CDS after the trial, indicating acceptability or perceived usefulness rather than demonstrated clinical effectiveness.

Across studies, reported sensitivity, negative predictive value, positive predictive value, and false-negative burden varied substantially, and prediction models were predominantly internally rather than externally validated. Key numerical findings are summarized in Table 6. 

**Table 6:** Key numerical findings from clinical and digital approaches to early recognition of pediatric sepsis Principal quantitative findings from the 13 included studies. Results were synthesized narratively because of substantial clinical and methodological heterogeneity. AUC/AUROC, area under the receiver operating characteristic curve; aHR, adjusted hazard ratio; aOR, adjusted odds ratio; CI, confidence interval; CDS, clinical decision support; CV, cross-validation; eST, electronic screening tool; LR+/LR-, positive/negative likelihood ratio; NLR, negative likelihood ratio; NNE, number needed to evaluate; NPV, negative predictive value; OOB, out-of-bag; PLR, positive likelihood ratio; PPV, positive predictive value; QI, quality improvement; SIRS, systemic inflammatory response syndrome; SPC, statistical process control

Study	Sample/Events	Principal Numerical Findings
Sepanski et al. [15]	n = 7,402; 38 severe sepsis	Sensitivity 97.4%; specificity 99.5%; PPV 48.7%; NPV 99.99%; AUC 0.984. False positives decreased 217→39, with 1 false negative in both versions. Median tool firing 11.1 h
Weiss et al. [16]	n = 6,925 screened; 706 cases by ≥1 definition	Both definitions: 301/706 (42.6%); κ 0.57 ± 0.02. Among 438 physician-diagnosed cases, 301 (69%) met consensus criteria. Regional agreement 31%-85%, P < .001
Lamping et al. [17]	n = 289 episodes; 56 sepsis	Training OOB-AUC 0.82; temporal validation AUC 0.78 (95% CI 0.70-0.87). At 100% sepsis identification, SIRS correctly classified 28%; Youden threshold: sepsis 84%, SIRS 61%; 3-fold CV AUC 0.75
Gomes et al. [18]	n = 19,912 screened; 90 sepsis	eST alone: sensitivity 86.7%, specificity 87.0%, PPV 2.94%, NPV 99.9%, LR+ 6.68, LR− 0.15. eST + all medical evaluations: sensitivity 90.0%, specificity 99.4%, PPV 42.0%, NPV 99.9%, LR+ 159.3, LR− 0.10
Ehwerhemuepha et al. [19]	n = 537,837 encounters; 295 severe sepsis	Severe-sepsis AUROC 0.990 (95% CI 0.985-0.995). Threshold 0.0028: sensitivity 84.5%, specificity 99.0%, PPV 6.31%, NPV 99.99%, NNE 16. At 99.5% specificity: sensitivity 71.1% (62.1-80.2)
Yan et al. [20]	n = 111,327 encounters; 414 shock cases across periods	Sensitivity 74.1% vs 72.5%; specificity 93.2% vs 94.0%; PPV 4.1% and NPV 99.9% in both periods. Shock without alert: 25.9% vs 27.5%
Gilholm et al. [21]	n = 3,473; 523 sepsis	32-item AUC 0.80 (0.78-0.82); 16-item AUC 0.80 (0.78-0.82). At sensitivity 90% (87-92): specificity 51%, PLR 1.83, NLR 0.20. Organ dysfunction AUC 0.84; shock AUC 0.84
Oddiri et al. [22]	QI bundle cohorts n = 41 baseline; 20, 18, 17, 13 thereafter	1-h bundle compliance 37% → 50% → 61% → 65% → 69%, P=.006. Provider-trigger alert response 67%→91%. Dashboard centerline 74%→87%, P=.002. Audit reported 0 missed coded severe-sepsis/shock cases
Li et al. [23]	n = 240; 86 shock by 2005 criteria	2024 criteria identified 80 sepsis and 49 shock. Overall κ 0.161; shock κ 0.630. Shock sensitivity 57.0%, specificity 100%, PPV 100%, NPV 80.6%
Dewan et al. [24]	n = 424 in-PICU sepsis-positive episodes	Overall sensitivity 92.5% (89.5-94.8); specificity 95.6% (95.2-96.0); PPV 46.0%; NPV 99.7%; LR+ 20.9; LR− 0.08. Chart-confirmed severe-sepsis sensitivity 73.3%. Timely 3-task bundle 18.6%→34.0%, P=.04
Scott et al. [25]	n = 3,843 registry patients; 932 severe sepsis	Arrival-to-recognition approximately 50→4 min. Recognition-to-antibiotic SPC median: 42.8 min Stat, 58.6 min Yellow. ICU care within 24 h 45%→34%. Mortality 0.9% (8/932). Missed-group arrival-to-recognition median 116 min
Sever et al. [26]	n = 492; 118 sepsis at presentation	Parent AUC 0.53, aOR 1.18 (0.89-1.58); nurse AUC 0.57, aOR 1.43 (1.10-1.87); physician AUC 0.63, aOR 1.61 (1.21-2.19). Baseline model AUC 0.75/0.66/0.70 across corresponding subgroups
Scott et al. [27]	n = 1,331 eligible; 979 intervention, 352 control	Concordant care 39.0% vs 38.9%, aOR 1.07 (0.61-1.88); shock aOR 1.12 (0.53-2.46); antibiotic timing aHR 0.85 (0.63-1.16). Prospective PPV 12%; CDS retained at 4/4 sites

Risk of Bias

Risk of bias and methodological quality were evaluated using design-specific tools: QUADAS-2 for diagnostic-accuracy and recognition studies, PROBAST+AI for prediction-model studies, ROBINS-I-informed domains for nonrandomized quality-improvement studies, and RoB 2 for the cluster randomized trial. Risk-of-bias assessments were used to inform interpretation of the evidence and were not used as an eligibility criterion. Overall judgments and the principal methodological concerns are summarized in Table 7.

**Table 7:** Overall methodological quality and risk-of-bias summary of included studies Overall judgments reflect the design-specific assessment framework applied to each study. For PROBAST+AI studies, model-development methodological quality and model-performance evaluation risk of bias are reported separately because these represent distinct assessments. PROBAST+AI, Prediction model Risk Of Bias ASsessment Tool plus Artificial Intelligence; QI, quality improvement; QUADAS-2, Quality Assessment of Diagnostic Accuracy Studies 2; RoB 2, revised Cochrane Risk of Bias tool; ROB, risk of bias; ROBINS-I, Risk Of Bias In Non-randomized Studies of Interventions

Study	Assessment Tool	Overall Judgment	Principal Methodological Concern
Sepanski et al. [15]	PROBAST+AI	Development quality: LOW; Evaluation ROB: HIGH	Limited events, extensive model refinement, and internal evaluation
Weiss et al. [16]	QUADAS-2	HIGH	Absence of a definitive gold standard and nonstandardized clinician diagnosis
Lamping et al. [17]	PROBAST+AI	Development quality: LOW; Evaluation ROB: SOME CONCERNS	Modest event numbers and internal temporal validation
Gomes et al. [18]	QUADAS-2	HIGH	Treatment-linked reference standard and incomplete independent verification
Ehwerhemuepha et al. [19]	PROBAST+AI	Development quality: LOW; Evaluation ROB: HIGH	Imperfect outcome labels and internal model evaluation
Yan et al. [20]	QUADAS-2	HIGH	Operational treatment-linked shock reference standard
Gilholm et al. [21]	PROBAST+AI	Development quality: LOW; Evaluation ROB: HIGH	Selected population and absence of independent external validation
Oddiri et al. [22]	ROBINS-I-informed	SERIOUS	Confounding from concurrent interventions and temporal changes
Li et al. [23]	QUADAS-2	HIGH	Reference-based cohort selection and non-independent comparator
Dewan et al. [24]	QUADAS-2	HIGH	Treatment-linked composite outcome and nonuniform reference verification
Scott et al. [25]	ROBINS-I-informed	SERIOUS	Uncontrolled QI design with multiple simultaneous interventions
Sever et al. [26]	QUADAS-2	HIGH	Selected screened population and imperfect sepsis reference standard
Scott et al. [27]	RoB 2	SOME CONCERNS	Potential post-randomization selection into the analytic cohort

Diagnostic-Accuracy and Prediction-Model Studies

Reference-standard concerns were identified in all six QUADAS-2 studies, although their nature differed. These included the absence of a definitive gold standard in Weiss et al. [16]; treatment-linked or non-independent reference standards in Gomes et al. [18], Yan et al. [20], and Dewan et al. [24]; reference-based cohort selection and use of a non-independent comparator in Li et al. [23]; and a selected screened population with an imperfect sepsis reference standard in Sever et al. [26].

PROBAST+AI assessments identified important limitations in prediction-model development and evaluation. Development quality was low for all four studies [15,17,19,21], reflecting concerns related to participant selection, event numbers, outcome definitions, model refinement, or analysis. During model-performance evaluation, Sepanski et al. [15], Ehwerhemuepha et al. [19], and Gilholm et al. [21] remained at high risk of bias. Lamping et al. [17] was judged to have some concerns because the model was evaluated only through internal temporal validation and the number of sepsis events was modest.

Domain-level QUADAS-2 assessments and PROBAST+AI model-development judgments are presented in Figure 2. 

**Figure 2:**
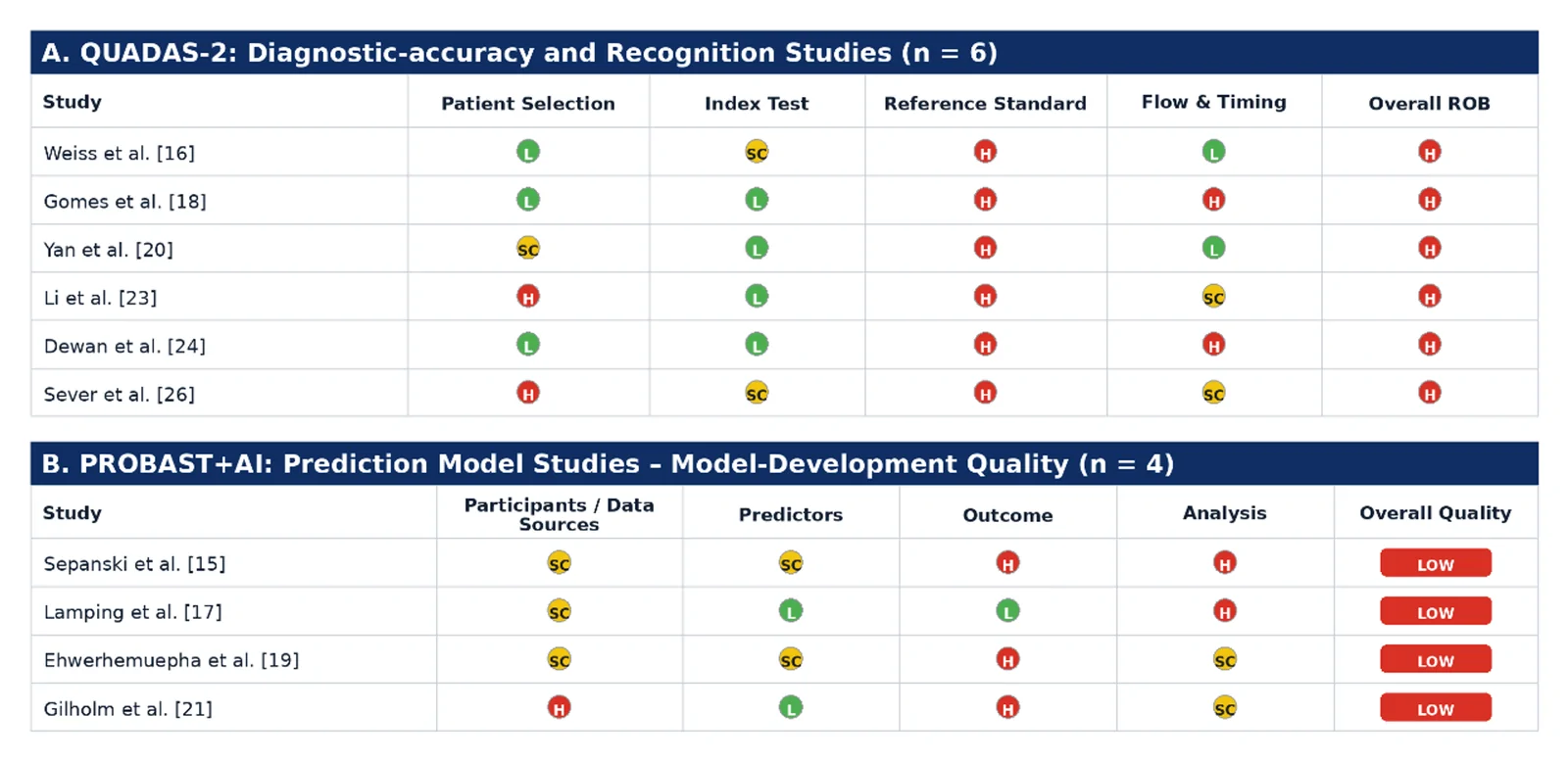
Domain-level assessment of diagnostic-accuracy studies and prediction-model development (A) QUADAS-2 judgments for diagnostic-accuracy and recognition studies [16,18,20,23,24,26]. (B) PROBAST+AI model-development methodological quality assessments for prediction-model studies [15,17,19,21]. For PROBAST+AI model development, the overall judgment refers to methodological quality rather than risk of bias; therefore, LOW indicates low methodological quality. Green indicates low risk or low concern, yellow indicates some concerns, and red indicates high risk or high concern. QUADAS-2, Quality Assessment of Diagnostic Accuracy Studies 2; PROBAST+AI, Prediction model Risk Of Bias ASsessment Tool plus Artificial Intelligence; ROB, risk of bias.

Model-Performance Evaluation and Intervention Studies

PROBAST+AI model-performance evaluation showed high risk of bias for three of the four prediction studies [15,19,21], with the study by Lamping et al. [17] judged to have some concerns. The main limitations were related to participant selection, outcome definitions, internal rather than external evaluation, and analytical concerns.

For nonrandomized interventions, studies by Oddiri et al. [22] and Scott et al. [25] were both judged to have a serious overall risk of bias using ROBINS-I-informed domains. Both were uncontrolled quality-improvement programs incorporating multiple concurrent interventions and temporal changes, limiting attribution of observed improvements specifically to the recognition intervention.

The stepped-wedge cluster randomized trial by Scott et al. was judged to have some concerns overall using RoB 2 [27]. The randomization process, outcome data, and reporting were generally robust; however, inclusion in the analytic cohort depended on clinician initiation of a sepsis evaluation after cluster-period allocation was known, creating potential post-randomization selection. The smaller-than-planned sample primarily affected precision rather than internal validity.

Domain-level PROBAST+AI model-performance, ROBINS-I-informed, and RoB 2 assessments are presented in Figure 3.

**Figure 3:**
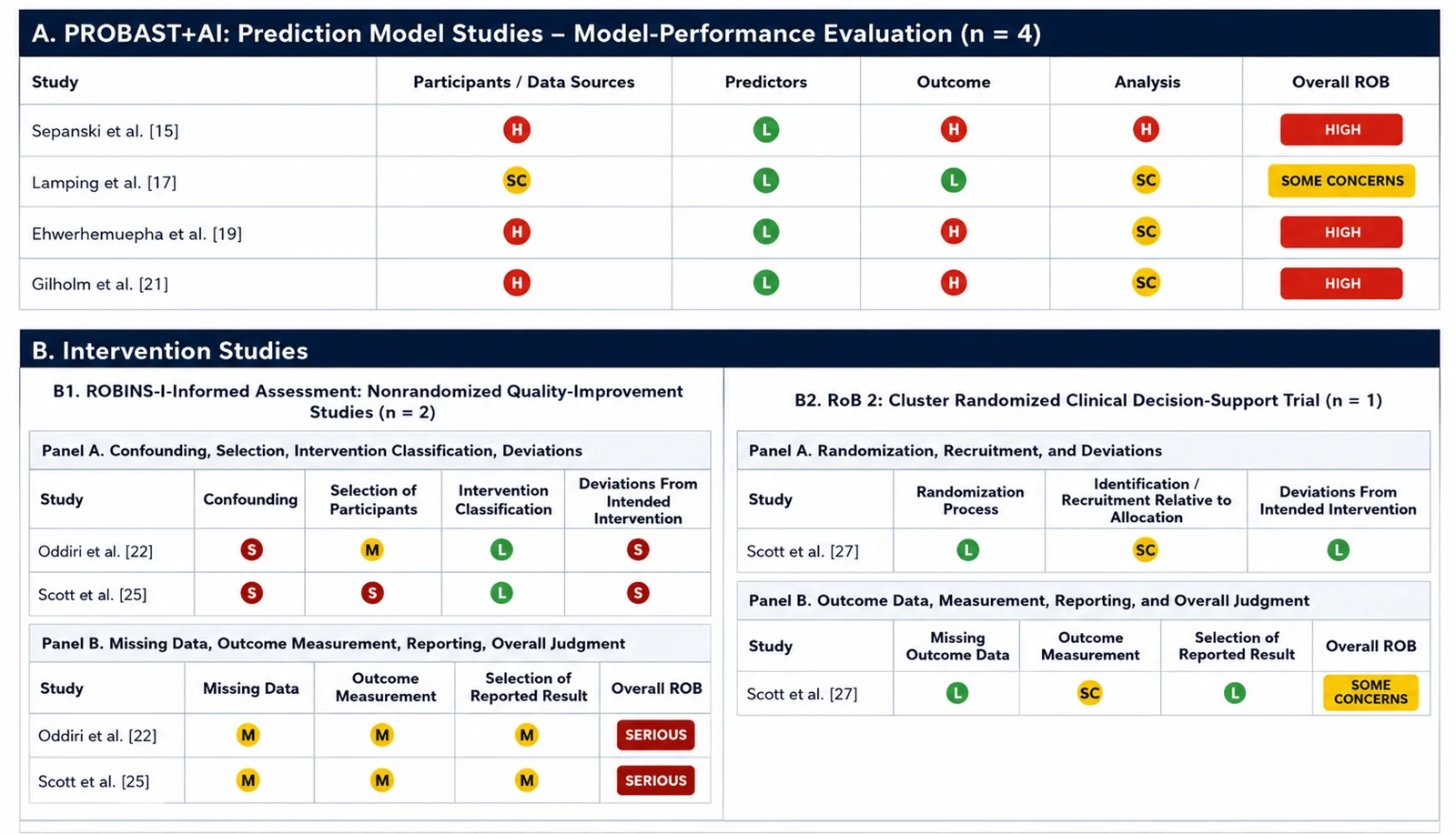
Domain-level assessment of prediction-model performance and intervention studies (A) PROBAST+AI model-performance risk-of-bias judgments for prediction-model studies [15,17,19,21]. (B) The intervention-study assessments: (B1) ROBINS-I-informed judgments for the nonrandomized quality-improvement studies [22,25], (B2) RoB 2 judgments for the cluster-randomized trial [27]. Green indicates low risk, yellow indicates some concerns, orange indicates moderate risk under ROBINS-I, and red indicates high or serious risk according to the applicable assessment tool. PROBAST+AI, Prediction model Risk Of Bias ASsessment Tool plus Artificial Intelligence; QI, quality improvement; RoB 2, revised Cochrane risk-of-bias tool for randomized trials; ROB, risk of bias; ROBINS-I, Risk Of Bias In Non-randomized Studies of Interventions

Discussion

Principal Findings and Interpretation

Across the 13 included studies, no single clinical or digital approach consistently provided reliable early recognition of pediatric sepsis. The clearest overall finding was that automated systems appeared most useful when combined with structured clinical assessment, although performance varied across settings, sepsis definitions, and reference standards. The evidence was drawn mainly from pediatric emergency departments, which were represented in nine studies, while four studies focused on PICU populations. Among the emergency-based studies, one also included pediatric inpatient units and another included urgent-care sites [15-27]. The evaluated approaches included electronic screening tools, clinical decision-support systems, machine-learning and prediction models, sepsis criteria, structured clinician assessment, human concern, and quality-improvement pathways.

Although several automated screening and prediction systems reported high sensitivity or negative predictive value, their safety cannot be judged from negative predictive value alone because this measure is strongly influenced by disease prevalence. Sensitivity, false-negative findings, and missed-case rates are therefore particularly important when assessing whether a recognition system can be used safely in routine care [15,18-21,24,25]. This concern was illustrated by Yan et al., and approximately one-quarter of hypotensive shock cases in their study occurred without an alert [20]. In Gomes et al.'s study, adding clinical evaluation to electronic screening increased sensitivity from 86.7% to 90.0% and positive predictive value from 2.94% to 42.0%, showing that clinician assessment substantially reduced false-positive classifications while providing a smaller improvement in true-case identification [18].

Strong statistical performance did not necessarily translate into reliable standalone clinical recognition. Sepanski et al. [15], Ehwerhemuepha et al. [19], and Dewan et al. [24] reported high sensitivity, discrimination, or negative predictive value for electronic approaches. Gilholm et al. found that the simplified 16-feature tool retained the same overall discrimination as the 32-item tool, with an AUC of 0.80 for both models [21]. However, when the threshold was selected to achieve 90% sensitivity, specificity fell to 51%, meaning that many children without sepsis would still screen positive. These findings suggest that automated recognition systems may be most useful for identifying children who require further assessment rather than independently determining sepsis status.

In populations where severe sepsis or septic shock is uncommon, a high negative predictive value may partly reflect the low number of true cases rather than the strength of the screening tool alone. A low positive predictive value may also persist despite apparently strong discrimination or specificity, as demonstrated by Ehwerhemuepha et al. [19]. In routine care, missed cases carry the greatest immediate safety concern because they may delay assessment and treatment. False-positive alerts are also important because they increase workload and alert fatigue, but their clinical importance depends on the burden and urgency of the action triggered by the alert. Evaluation should therefore consider false-positive alerts, missed cases, workload, escalation requirements, and the clinical consequences of acting on or withholding a response to an alert.

Recognition Strategies and Sepsis Definitions

The evidence suggests a potentially important role for hybrid recognition strategies that combine electronic surveillance with structured clinical assessment. In Gomes et al., the addition of clinical evaluation increased specificity from 87.0% to 99.4% and positive predictive value from 2.94% to 42.0%, while sensitivity increased more modestly from 86.7% to 90.0% [18]. This indicates that clinician assessment mainly improved efficiency by reducing false-positive classifications, while also producing a smaller improvement in true-case identification. Dewan et al. similarly used a two-stage clinical decision-support pathway in which an electronic trigger was followed by nurse assessment of perfusion [24]. In contrast, subjective concern alone appeared insufficient. Sever et al. found limited discriminatory ability for parental, nursing, and physician concern, with little improvement when concern was added to baseline clinical models [26]. Taken together, these findings suggest that neither automation nor subjective clinical assessment is universally adequate in isolation and that combining physiologic information, structured surveillance, and clinician interpretation may provide a more clinically realistic approach.

Oruganti et al. identified substantial heterogeneity across pediatric emergency-department recognition methods and found no single consistently reliable identification strategy [6]. Reviews of pediatric prediction technologies and digital sepsis solutions have similarly reported variation in populations, prediction horizons, outcomes, and clinical integration, with many models evaluated retrospectively or within single institutions [7,8]. The present review extends these observations by offering a unified synthesis that integrates sepsis criteria, clinical assessment, electronic alerts, prediction technologies, and implementation pathways within a single comparative framework.

Variation in sepsis definitions and reference standards further complicated comparisons across studies. Weiss et al. reported only moderate agreement between clinician diagnosis and consensus criteria, with substantial geographic variation [16], while Li et al. found considerable disagreement between the 2005 pediatric consensus criteria and the newer 2024 Phoenix criteria [23]. The introduction of the Phoenix Sepsis Score represents a shift toward defining pediatric sepsis through infection-associated organ dysfunction [3], but criteria designed to classify established sepsis do not necessarily serve the same purpose as tools intended to recognize children at an earlier stage of deterioration. Some apparent false-positive alerts may therefore have reflected a temporal mismatch between the recognition tool and the reference standard. An early-warning system may identify evolving deterioration before the child meets criteria for established sepsis-related organ dysfunction. Its performance should be evaluated using time-linked outcomes that distinguish an unnecessary alert from an early alert that precedes later clinical deterioration. Consequently, disagreement between an early-warning system and an organ-dysfunction-based definition should not automatically be interpreted as evidence of poor clinical utility.

Reference-standard limitations were also reflected in the risk-of-bias assessment. All six studies evaluated using QUADAS-2 had high overall risk of bias, with recurrent concerns related to treatment-linked definitions, clinician-dependent classification, non-independent comparators, and selected populations [16,18,20,23,24,26]. These methodological differences can directly influence apparent sensitivity and specificity and limit meaningful comparison between recognition systems.

Prediction-model studies demonstrated similar challenges. Lamping et al. [17] and Ehwerhemuepha et al. [19] highlighted the potential of EHR-integrated data-driven models. Gilholm et al. found that a simplified screening framework retained discrimination comparable to the original model [21]. However, PROBAST+AI assessment identified low model-development quality across all four prediction studies [15,17,19,21] and high risk of bias in model-performance evaluation in three studies [15,19,21]. The reliance on internal rather than independent external evaluation remains an important barrier to wider clinical adoption. Performance within the development environment may not be maintained across hospitals with different patient populations, documentation practices, disease prevalence, electronic systems, and clinical workflows. High AUROC values should therefore not be interpreted as evidence of broad clinical generalizability without external validation.

Clinical Implications and Future Directions

From a clinical perspective, electronic alerts, prediction models, and structured screening systems appear most appropriately positioned as decision-support tools rather than replacements for clinician judgment. The preferred balance between sensitivity and specificity depends on the clinical response triggered by the system. An alert that prompts a low-burden action, such as repeat bedside assessment, may reasonably prioritize sensitivity. In contrast, an alert that directly initiates antibiotics, fluid resuscitation, or escalation of care may require greater specificity. The appropriate threshold must therefore reflect the intended action, local disease prevalence, staffing, digital infrastructure, and available escalation pathways.

The implementation studies reinforce the importance of this clinical context. Oddiri et al. [22], Dewan et al. [24], and Scott et al. [25] reported improvements in recognition time, bundle compliance, completion of recommended assessment tasks, alert-response compliance, and, in some settings, the timing of antibiotics or fluid administration following implementation of structured pathways or electronic support. However, the nonrandomized quality-improvement programs often introduced several interventions simultaneously, including education, order sets, paging systems, dashboards, audit and feedback, and workflow redesign [22,25]. Both nonrandomized intervention studies were judged to have a serious overall risk of bias, making it difficult to isolate the effect of the recognition system itself from accompanying changes in clinical practice.

This issue was highlighted by the stepped-wedge cluster randomized trial by Scott et al. [27]. Despite implementing predictive clinical decision support across participating sites, Scott et al. found no significant improvement in guideline-concordant care, progression to hypotensive shock, or time to antibiotics [27]. The contrast between favorable process measures in observational quality-improvement studies and neutral findings in the randomized trial shows that improved recognition or implementation does not necessarily translate into better patient outcomes.

Future research should therefore prioritize prospective, multicenter external validation across healthcare environments that differ meaningfully from model-development settings. Direct comparisons of automated, clinician-based, and hybrid strategies within the same populations would help determine whether electronic surveillance provides incremental benefit beyond structured clinical assessment and whether clinician review can reduce false-positive alerts without increasing missed sepsis.

Evaluation also needs to move beyond discrimination metrics such as AUROC. Future studies need to evaluate three outcome domains: model performance, including calibration, clinically relevant thresholds, false-positive burden, false-negative cases, and missed or delayed recognition; care processes, including time to escalation, time to antibiotics where appropriate, unnecessary treatment, and bundle completion; and patient outcomes, including organ dysfunction, ICU admission, length of stay, and mortality. Randomized or well-controlled implementation studies are particularly important for distinguishing the effect of recognition systems from concurrent education and workflow changes.

Finally, the clinical purpose of each recognition system needs to be explicitly defined. Screening for early risk, predicting septic shock, identifying established organ dysfunction, prompting bedside reassessment, and supporting bundle completion represent related but distinct clinical goals. Future research needs to align decision thresholds, reference standards, and primary endpoints with the specific clinical action that the system is intended to trigger. Transparent, purpose-driven reference standards are also essential for maintaining cross-study comparability as pediatric sepsis definitions continue to evolve.

A strength of this review was its broad evaluation of clinical screening, electronic alerts, machine-learning models, sepsis criteria, clinician assessment, structured pathways, quality-improvement interventions, and randomized decision support within a single synthesis. The use of design-specific appraisal tools-QUADAS-2, PROBAST+AI, ROBINS-I-informed assessment, and RoB 2-also allowed methodological concerns to be interpreted according to the different study designs rather than through a single generic quality framework.

Limitations

This review has several limitations. It was retrospectively registered because searching and initial screening began before registration, and subsequent refinement of the eligibility criteria may have introduced decisions that were not fully prespecified. Four potentially relevant reports could not be retrieved for full-text assessment. The formal search was limited to PubMed/MEDLINE and Scopus, so relevant studies indexed exclusively in other databases may have been missed. In addition, the original literal supplementary PubMed and Scopus query strings were not retained, which limits exact reproducibility despite the preserved search framework and database yields. Substantial heterogeneity across patient populations, study settings, sepsis definitions, reference standards, thresholds, prediction horizons, and clinical outcomes precluded formal meta-analysis and limited direct comparisons between recognition tools. Because most studies were conducted in tertiary pediatric centers in high-resource countries, generalizability to community emergency departments and resource-limited settings remains uncertain. Treatment-linked or otherwise imperfect reference standards, reliance on internal model validation, and confounding in nonrandomized studies further reinforce the need for cautious interpretation of reported recognition performance and care-process outcomes.

## Conclusions

Clinical screening, electronic alerts, prediction models, and decision-support systems can assist the early recognition of pediatric sepsis, but current evidence does not identify a universally superior approach. Automated systems appear most promising when integrated with structured clinical assessment, including formal bedside screening tools and direct clinician evaluation. Their performance remains influenced by sepsis definitions, thresholds, disease prevalence, and healthcare context. Given the heterogeneity and methodological limitations of the available evidence, these tools should support rather than replace clinical judgment. Further multicenter validation and outcome-focused trials are needed to determine which strategies provide reliable and clinically meaningful benefits across diverse pediatric settings.

## References

[1] Fleischmann-Struzek C, Goldfarb DM, Schlattmann P, Schlapbach LJ, Reinhart K, Kissoon N (2018). The global burden of paediatric and neonatal sepsis: a systematic review. Lancet Respir Med.

[2] Weiss SL, Fitzgerald JC (2024). Pediatric sepsis diagnosis, management, and sub-phenotypes. Pediatrics.

[3] Schlapbach LJ, Watson RS, Sorce LR (2024). International consensus criteria for pediatric sepsis and septic shock. JAMA.

[4] Weiss SL, Peters MJ, Oczkowski SJ (2026). Surviving Sepsis Campaign international guidelines for the management of sepsis and septic shock in children 2026. Pediatr Crit Care Med.

[5] Menon K, Schlapbach LJ, Akech S (2022). Criteria for pediatric sepsis-a systematic review and meta-analysis by the Pediatric Sepsis Definition Taskforce. Crit Care Med.

[6] Oruganti S, Evans J, Cromarty T, Javaid A, Roland D (2022). Identification of sepsis in paediatric emergency departments: a scoping review. Acta Paediatr.

[7] Sanchez-Pinto LN, Del Pilar Arias López M, Scott H (2024). Digital solutions in paediatric sepsis: current state, challenges, and opportunities to improve care around the world. Lancet Digit Health.

[8] Tennant R, Graham J, Kern J, Mercer K, Ansermino JM, Burns CM (2024). A scoping review on pediatric sepsis prediction technologies in healthcare. NPJ Digit Med.

[9] Driver B, Babl FE, Cheng D (2026). Alerts and alarms-can electronic medical records help children and adolescents survive sepsis?. J Paediatr Child Health.

[10] Page MJ, McKenzie JE, Bossuyt PM (2021). The PRISMA 2020 statement: an updated guideline for reporting systematic reviews. BMJ.

[11] Whiting PF, Rutjes AW, Westwood ME (2011). QUADAS-2: a revised tool for the quality assessment of diagnostic accuracy studies. Ann Intern Med.

[12] Moons KG, Damen JA, Kaul T (2025). PROBAST+AI: an updated quality, risk of bias, and applicability assessment tool for prediction models using regression or artificial intelligence methods. BMJ.

[13] Sterne JA, Hernán MA, Reeves BC (2016). ROBINS-I: a tool for assessing risk of bias in non-randomised studies of interventions. BMJ.

[14] Sterne JA, Savović J, Page MJ (2019). RoB 2: a revised tool for assessing risk of bias in randomised trials. BMJ.

[15] Sepanski RJ, Godambe SA, Mangum CD, Bovat CS, Zaritsky AL, Shah SH (2014). Designing a pediatric severe sepsis screening tool. Front Pediatr.

[16] Weiss SL, Fitzgerald JC, Maffei FA (2015). Discordant identification of pediatric severe sepsis by research and clinical definitions in the SPROUT international point prevalence study. Crit Care.

[17] Lamping F, Jack T, Rübsamen N (2018). Development and validation of a diagnostic model for early differentiation of sepsis and non-infectious SIRS in critically ill children - a data-driven approach using machine-learning algorithms. BMC Pediatr.

[18] Gomes S, Wood D, Ayis S, Haliasos N, Roland D (2021). Evaluation of a novel approach to recognising community-acquired paediatric sepsis at ED triage by combining an electronic screening algorithm with clinician assessment. Emerg Med J.

[19] Ehwerhemuepha L, Heyming T, Marano R (2021). Development and validation of an early warning tool for sepsis and decompensation in children during emergency department triage. Sci Rep.

[20] Yan AP, Zipursky AR, Capraro A, Harper M, Eisenberg M (2022). Pediatric emergency department sepsis screening tool accuracy during the COVID-19 pandemic. Pediatrics.

[21] Gilholm P, Gibbons K, Lister P (2023). Validation of a paediatric sepsis screening tool to identify children with sepsis in the emergency department: a statewide prospective cohort study in Queensland, Australia. BMJ Open.

[22] Oddiri U, Propper G, Brill P, Reid B, Giarraputo D, Milana C (2023). Early identification of severe sepsis in pediatric patients using an electronic alert system. Hosp Pediatr.

[23] Li W, Ge H, Zhang J (2025). Comparison of 2005 and 2024 diagnostic criteria for early identification of pediatric sepsis and septic shock in PICU patients: a prospective cohort study. Front Pediatr.

[24] Dewan M, Vidrine R, Zackoff M (2020). Design, implementation, and validation of a pediatric ICU sepsis prediction tool as clinical decision support. Appl Clin Inform.

[25] Scott HF, Kempe A, Deakyne Davies SJ (2020). Managing diagnostic uncertainty in pediatric sepsis quality improvement with a two-tiered approach. Pediatr Qual Saf.

[26] Sever Z, Schlapbach LJ, Gilholm P (2023). Impact of parental and healthcare professional concern on the diagnosis of pediatric sepsis: a diagnostic accuracy study. Front Pediatr.

[27] Scott HF, Sevick CJ, Colborn KL (2025). Clinical decision support for septic shock in the emergency department: a cluster randomized trial. Pediatrics.

